# PI3K/AKT pathway regulates E-cadherin and Desmoglein 2 in aggressive prostate cancer

**DOI:** 10.1002/cam4.463

**Published:** 2015-05-29

**Authors:** Alison G Barber, Mireia Castillo-Martin, Dennis M Bonal, Angela J Jia, Benjamin A Rybicki, Angela M Christiano, Carlos Cordon-Cardo

**Affiliations:** 1 Department of Genetics and Development, Columbia UniversityNew York City, New York; 2 Department of Pathology, Icahn School of Medicine at Mount SinaiNew York City, New York; 3 Herbert Irving Comprehensive Cancer Center, Columbia UniversityNew York City, New York; 4 Department of Public Health Sciences, Henry Ford Health SystemDetroit, Michigan; 5 Department of Dermatology, Columbia UniversityNew York City, New York; 6 Department of Pathology and Cell Biology, Columbia UniversityNew York City, New York; 7 Department of Urology, Columbia UniversityNew York City, New York

**Keywords:** Desmoglein 2, E-cadherin, PI3K/AKT pathway, prognostic markers, prostate cancer

## Abstract

Reduced expression of both classical and desmosomal cadherins has been associated with different types of carcinomas, including prostate cancer. This study aims to provide a comprehensive view of the role and regulation of cell–cell adhesion in prostate cancer aggressiveness by examining the functional implications of both E-cadherin and Desmoglein 2 (DSG2). E-cadherin expression was first examined using immunofluorescence in 50 normal prostate tissues and in a cohort of 414 prostate cancer patients. Correlation and survival analyses were performed to assess its clinical significance. In primary prostate cancer patients, reduced expression of both E-cadherin and DSG2 is significantly associated with an earlier biochemical recurrence. Transgenic DU145 E-cadherin knockdown and constitutively active AKT overexpression lines were generated. Functional implications of such genetic alterations were analyzed in vitro and in vivo, the latter by using tumorigenesis as well as extravasation and metastatic tumor formation assays. We observed that loss of E-cadherin leads to impaired primary and metastatic tumor formation in vivo, suggesting a tumor promoter role for E-cadherin in addition to its known role as a tumor suppressor. Activation of AKT leads to a significant reduction in E-cadherin expression and nuclear localization of Snail, suggesting a role for the PI3K/AKT signaling pathway in the transient repression of E-cadherin. This reduced expression may be regulated by separate mechanisms as neither the loss of E-cadherin nor activation of AKT significantly affected DSG2 expression. In conclusion, these findings illustrate the critical role of cell–cell adhesion in the progression to aggressive prostate cancer, through regulation by the PI3K pathway.

## Introduction

Prostate cancer is the most frequently diagnosed cancer in American men and the second leading cause of cancer death. Importantly, there is only a 29% 5-year survival rate for metastatic prostate cancer 1. In order to metastasize, a cancer cell must detach itself from the primary tumor and adopt migratory properties which allow it to first invade the surrounding stroma, then to reach vascular structures, and finally to form a tumor at a distant site 2. Alterations in cell–cell adhesion are essential for these key stages of the metastatic pathway, as they allow for the initial detachment and the later metastatic tumor formation.

There are two subtypes of anchoring junctions that differ in the cytoskeletal filaments to which they attach: adherens junctions and desmosomes 3,4. These junctions are interdependent with respect to their assembly and regulation, and the formation of adherens junctions has been shown to precede that of desmosomes in both early development and in the de novo formation of anchoring junctions 5–7. Central to these junctions are cadherins 8–10. This study focuses on E-cadherin, the predominant cadherin in adherens junctions of epithelial tissues, and Desmoglein 2 (DSG2), a ubiquitous desmosomal cadherin isoform 11,12.

Downregulation of E-cadherin, which can be caused by loss of heterozygosity (LOH), mutations, or transcriptional silencing, is a common feature of a variety of cancers, including prostate cancer 13,14. We have recently reported that reduced expression of DSG2 is an independent prognostic factor in primary prostate cancer patients 15. However, re-expression of adherens junctions is often found in metastatic tumors including those of the prostate 16–18. This demonstrates a need for maintaining a dynamic regulation of cell–cell adhesion in order to survive as a metastatic cancer cell. The loss of E-cadherin expression is an underlying hallmark of both epithelial-mesenchymal transition (EMT) and tumor progression 19,20. Additionally, the loss of E-cadherin has been linked to transcriptional repression via effectors of EMT such as Snail 21–25. Furthermore, the PI3K/AKT signaling pathway has been linked to EMT in cancer 26–28. AKT may have a role in the loss of E-cadherin expression as it has been shown that AKT activation leads to the downregulation of E-cadherin expression and the upregulation of Snail expression 29,30. Interestingly, other studies suggest that desmosome formation may be affected by AKT activity as well 29.

In this study, we show that reduced expression of both E-cadherin and DSG2 is observed in primary prostate cancer, and that this reduced expression is significantly associated with a shorter biochemical recurrence (BCR)-free survival, rendering both classical and desmosomal cadherins as markers of poor prognosis in patients with prostate cancer. Additionally, the loss of E-cadherin does not result in the reciprocal loss of DSG2 in prostate cancer cells in vitro. Interestingly, we provide functional evidence for the role of E-cadherin in promoting the formation of primary and metastatic tumors in vivo, and we demonstrate that transient repression of E-cadherin in prostate cancer cells may be mediated by the PI3K/AKT signaling pathway as a possible consequence of increased Snail activity.

## Material and Methods

### Mouse experiments

All assays were performed on 5–6-week-old male NOD.CB17-Prkdc^Scid^ (NOD/SCID) mice from Jackson Laboratories (The Jackson Laboratories, Bar Harbor, ME). Animal use and care followed institutional guidelines established by the Columbia University Institutional Animal Care and Use Committee.

### Generation of stable cell lines

The stable E-cadherin knockdown (EcadKD) cell line was generated using a pSMP retroviral shRNAmir construct targeting E-cadherin, purchased from Open Biosystems (Thermo Fisher Scientific, Huntsville, AL). The stable myristoylated AKT HA-tagged (MAH) cell line was generated using a pLNCX retroviral construct containing myristoylated HA-tagged AKT1 purchased from Addgene (Cambridge, MA). Phoenix2™-Ampho cells (Allele Biotechnology, San Diego, CA) were used as viral vehicles. Arrest-In™ (Open Biosystems/Thermo Fisher Scientific, Huntsville, AL) and FuGENE 6® (Roche Applied Sciences, Indianapolis, IN) were used as transfection reagents for the EcadKD and MAH cells, respectively. DU145 cell line was infected three times via a direct transfer of filtered media containing 10 *μ*g/mL Polybrene (Millipore, Billircia, MA) from the transfected Phoenix2™-Ampho cells. We used Puromycin and Geneticin (Invitrogen, Carlsbad, CA) as selective agents for EcadKD cells and MAH cells, respectively.

### RNA and protein isolation

Cells grown 4 days past confluence were pelleted and RNA was harvested using the RNeasy Mini Kit and QIAshredder following the manufacturer’s protocol (Qiagen, Valencia, CA).

To isolate protein, 200 *μ*L of RIPA buffer (50 mmol/L Tris-HCl, pH 7.4, 150 mmol/L NaCl, 1% NP-40, 0.5% Sodium deoxycholate, 0.1% SDS) containing a protease inhibitor cocktail (Complete, EDTA-free, Roche Diagnostics, Indianapolis, IN) was added to cells on ice. Cells were then scraped and collected, and the protein lysate was obtained. Protein concentration was determined using the Bio-Rad Protein Assay (Bio-Rad, Hercules, CA).

### qRT-PCR

First strand cDNA was made using Oligo dT and the SuperScript® III First-Strand Synthesis System (Invitrogen) following the manufacturer’s instructions. qRT-PCR was performed on a Stratagene Mx3005P machine and analyzed using Stratagene MxPro QPCR software (Stratagene, Santa Clara, CA). All reactions were performed using QuantiTect™ SYBR® Green PCR Master Mix (Qiagen), All samples were run in quadruplicate, and were normalized against *β*-actin. Primers are summarized in Table S1.

### Antibodies

Antibodies corresponded to anti-E-cadherin (mouse monoclonal (clone HECD-1); Invitrogen); anti-DSG2 (mouse monoclonal (clone DG3.10); Fitzgerald, Acton, MA); anti-*β*-actin (mouse monoclonal [clone AC-74]; Sigma-Aldrich, St. Louis, MI); anti-HA-tag (rabbit monoclonal [clone C29F4]; Cell Signaling, Danvers, MA); anti-Snail (rabbit polyclonal; Abcam, Cambridge, MA); anti-pAKT(Ser473) (rabbit monoclonal (clone 736E11); Cell Signaling); and anti-CK8/18 (guinea pig polyclonal; Progen, Heidelberg, Germany).

### Western blot analysis

Protein lysates were separated by 4–20% Tris-HCl SDS-PAGE. Gel transfer to a nitrocellulose membrane was conducted using the iBlot® gel transfer system (Invitrogen). Membranes were blocked and incubated with primary antibody, followed by incubation in HRP conjugated secondary antibody (GE Healthcare, Little Chalfont, UK). Membrane was treated with ECL Plus Western Blotting Detection reagent (GE Healthcare) and visualized on Amersham Hyperfilm™ ECL (GE Healthcare). Quantification of the bands was performed using the Quantity One 1-D Analysis Software (Bio-Rad Laboratories/Life Science Research, Hercules, CA), and the number corresponding to the expression relative to the *β*-actin band are displayed below each band.

### In vivo tumorigenesis assay

1 × 10^6^ cells were subcutaneously injected with 200 *μ*L of the 1:1 cell-Matrigel suspension (BD Biosciences, Franklin Lakes, NJ) in NOD/SCID mice. Each mouse was injected with all three cell lines of interest, distributed as illustrated in Figure S1: DU145 cells in the upper-left flank, MAH cells in the upper-right flank and EcadKD cells in the lower-right flank. Tumors were allowed to form for 8 weeks at which point the animals were killed. Tumors were collected, weighed, and tumor volume was assessed via caliper measurement. Tumors were embedded in OCT (Sakura Finetek, Torrance, CA), snap frozen, and 5 *μ*m sections were used for immunofluorescence (IF) analysis. Two independent trials of the assay were performed, including six mice for the first trial and eight mice for the second trial.

### In vivo extravasation and metastatic tumor colony formation

1 × 10^6^ cells in a total volume of 100 *μ*L were injected into the lateral tail vein of NOD/SCID mice. Animals were killed 8 weeks after the injection and their lungs were collected, formalin fixed, and paraffin embedded. Six 5 *μ*m tissue sections separated by 100 *μ*m were stained with hematoxylin and eosin (H&E). Tumors in each lung section were counted by a pathologist (MCM). Tumors found exclusively in one tissue section were counted, whereas tumors found in multiple consecutive sections were only counted in the tissue section in which they first appeared. To account for extravasation, only metastatic tumor colonies found in the lung parenchyma were counted, not intravascular colonies. Two trials of the experiment were performed. Eight mice for each experimental group were used in the first trial. For the second trial five mice were used for the DU145 injections, nine mice were used for the MAH injections, and seven mice were used for the EcadKD injections.

### Immunofluorescence analysis of cell lines, frozen tissues, and formalin-fixed paraffin embedded (FFPE) tissue microarray (TMA) sections

Cell lines were grown on glass coverslips (Fisher, Pittsburgh, PA) and were treated in the same manner as slides of frozen tissue sections. Slides/coverslips were fixed and permeabilized and IF was performed using the same protocol as for FFPE tissues.

Tissue microarrays (TMAs) were built generating triplicate cores from 414 radical prostatectomy cases as previously described 15, which included 414 tumors and 50 adjacent histologically normal prostate samples. Five-micrometer sections were deparaffinized and submitted to antigen retrieval in citrate buffer, pH 6.0. They were incubated in blocking serum followed by primary antibody. Then, secondary antibodies either Alexa Fluor® 594 or Alexa Fluor® 488 (Invitrogen) were used and slides were mounted using VECTASHIELD® mounting medium with DAPI (Vector Laboratories, Burlingame, CA). TMAs were scored by determining the percentage of tumor cells with immunoreactivity for the protein of interest per tissue core. The average values of the representative cores from each patient sample were then used for statistical analyses. Clinico-pathological features of the 414 patients included in this study are summarized in Table S2.

### Documentation of biomarker suitability

Table S3 summarizes the biomarker evaluation in the human samples following the REMARK guidelines for prognostic markers 31.

### Statistical analysis

For in vitro and in vivo based assays, experimental data is expressed as mean ± SD; statistical analysis was performed using a Student’s *t*-test. Fisher’s exact test was used for the analysis of categorical data. For TMA-based assays, the Student’s *t*-test was used to compare the expression of the markers in primary prostate cancer and adjacent normal prostate tissue. Spearman’s rank correlation was used to analyze correlations between markers and clinico-pathological features. BCR-free survival was analyzed using Kaplan–Meier survival curves, and compared using the log-rank test. BCR was defined as a postsurgery undetectable PSA reading followed by two consecutive detectable (>0.2 ng/mL) rising PSA levels four weeks or more postsurgery 32. Statistical analyses were conducted using SPSS v20.0 (IBM, Chicago, IL). A two-sided *P* ≤ 0.05 was considered statistically significant.

## Results

### E-cadherin and DSG2 are critical prognostic markers in primary prostate cancer

**Figure 1:**
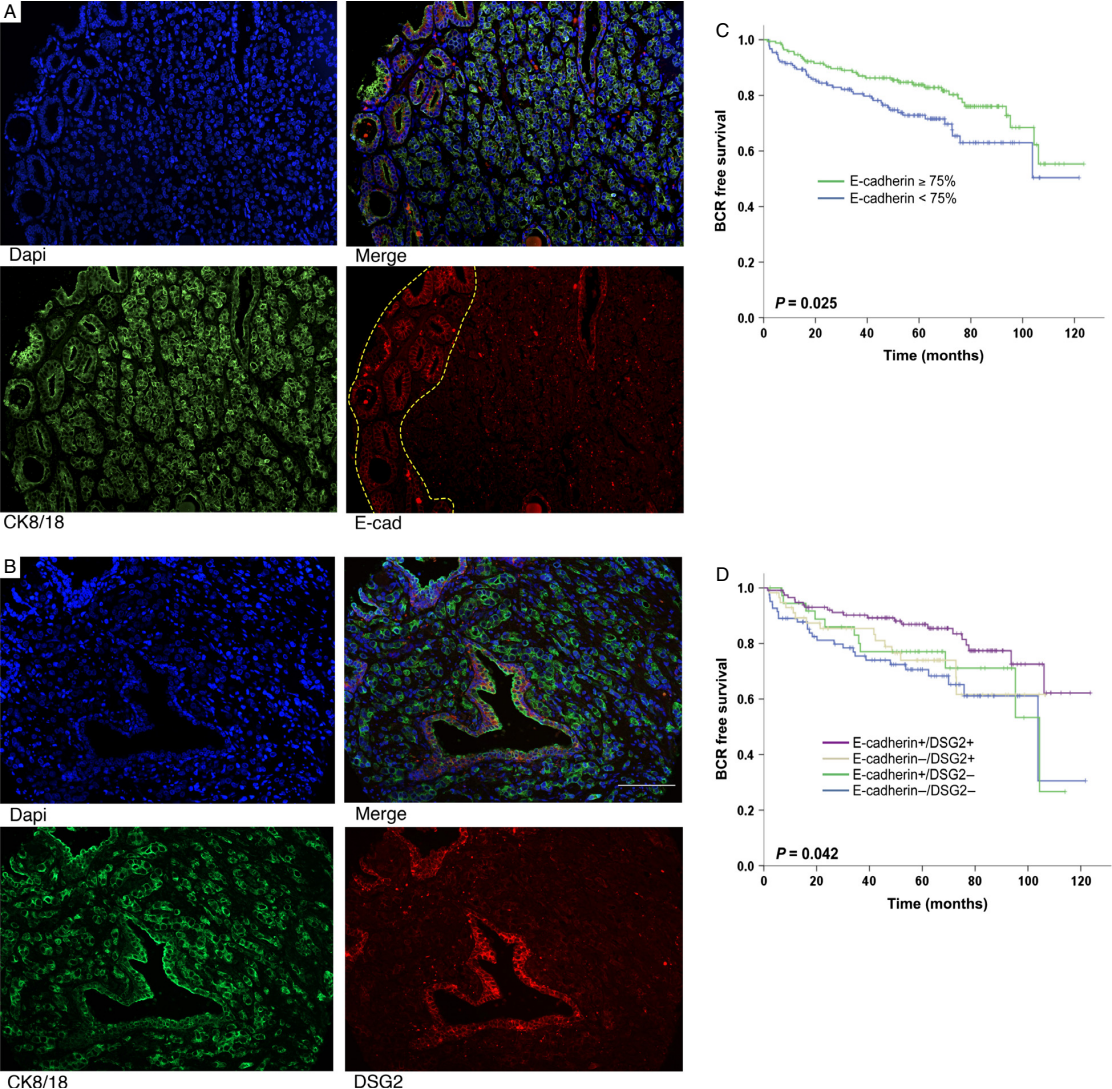
DSG2 expression is preserved in a DU145 cell line stably expressing an shRNAmir-E-cadherin construct. (A) qRT-PCR analysis shows that the EcadKD cell line has a 99.6% reduction in E-cadherin mRNA expression. (B) Representative western blot analysis displays a significant reduction in E-cadherin expression in the EcadKD line. (C) Immunofluorescence (IF) analysis confirms this reduction in E-cadherin protein expression. (D) qRT-PCR analysis shows only a slight reduction in *DSG2* mRNA expression in the EcadKD cell line, which is confirmed at the protein level both by (E) western blot and (F) IF analysis. Scale bar corresponds to 200 *μ*m.

We first examined E-cadherin expression in a cohort of 414 patients with primary prostate cancer, for whom DSG2 expression had already been reported 15. Clinico-pathological characteristics of these patients are summarized in Table S2. A significant decrease in E-cadherin expression was found in prostate cancer when compared with adjacent histologically normal prostate glands (Table1A), consistent with previously reported studies 13,33. Notably, cell border expression of E-cadherin was generally high in well-differentiated areas of the tumor, whereas it was much lower in poorly differentiated areas (Fig.1A). As we previously reported 15, there was a significant decrease in DSG2 expression (Fig.1B), with a strong correlation with E-cadherin expression (Table1B).

**Table 1:** Classical and desmosomal cadherin expression in normal prostate and prostate cancer. Correlation with clinico-pathological features of prostate cancer

	Prostate cancer	Normal glands	*P*-value^1^
E-cadherin			
Mean	69.2	87.2	0.0003
SD	20.2	3.6	
Median	75.0	88.0	
Interquartile range	60.0–83.3	85.0–90.0	

		PSA	Gleason score	TNM stage	E-cadherin	DSG2
PSA	Correlation	1.000	0.312^2^	0.256^2^	−0.072	−0.161^2^
	*Sig. (2-tailed)*	–	0.000	0.000	0.201	0.004
	*N*	410	400	410	317	316
Gleason score	Correlation		1.000	0.363^2^	−0.109	−0.160^2^
	*Sig. (2-tailed)*		–	0.000	0.054	0.005
	*N*		402	402	314	313
TNM stage	Correlation			1.000	−0.083	−0.084
	*Sig. (2-tailed)*			–	0.139	0.135
	*N*			412	319	318
E-cadherin	Correlation				1.000	0.498^2^
	*Sig. (2-tailed)*				–	0.000
	*N*				321	292
DSG2	Correlation					1.000
	*Sig. (2-tailed)*					–
	*N*					320

1 *P*-value determined using Student’s *t*-test.

2 Correlation is significant at the 0.01 level (two-tailed).

We observed negative correlations between E-cadherin expression and all clinico-pathological features examined (Table1B), including characteristics most commonly associated with aggressive prostate cancer 34. To examine the prognostic implication of E-cadherin loss, we used a cut-off value of 75% E-cadherin expression, as this was the median expression value observed for E-cadherin in our tumor cohort 15. Importantly, none of the adjacent normal prostate specimens examined displayed an expression of E-cadherin lower than 75%. Notably, patients whose prostate tumors showed an E-cadherin-positive phenotype (defined by having ≥75% E-cadherin expression) had a significantly longer recurrence-free survival than those expressing <75% E-cadherin (Fig.1C; *P *= 0.025). Furthermore, analyses of both E-cadherin and DSG2 expression together showed that loss of both markers was significantly associated with a worse prognosis (Fig.1D, *P* = 0.041), but DSG2 loss was more important than E-cadherin loss. However, multivariate analyses revealed that E-cadherin, unlike DSG2, was not an independent factor of BCR (*P *= 0.122). Taken together, these results indicate that reduced expression of both E-cadherin and DSG2 is significantly associated with BCR in prostate cancer, but that DSG2 alone, as previously reported by our group 15 may be the most useful prognostic marker.

### Formation of desmosomes and adherens junctions are independent processes in prostate cancer

**Figure 2:**
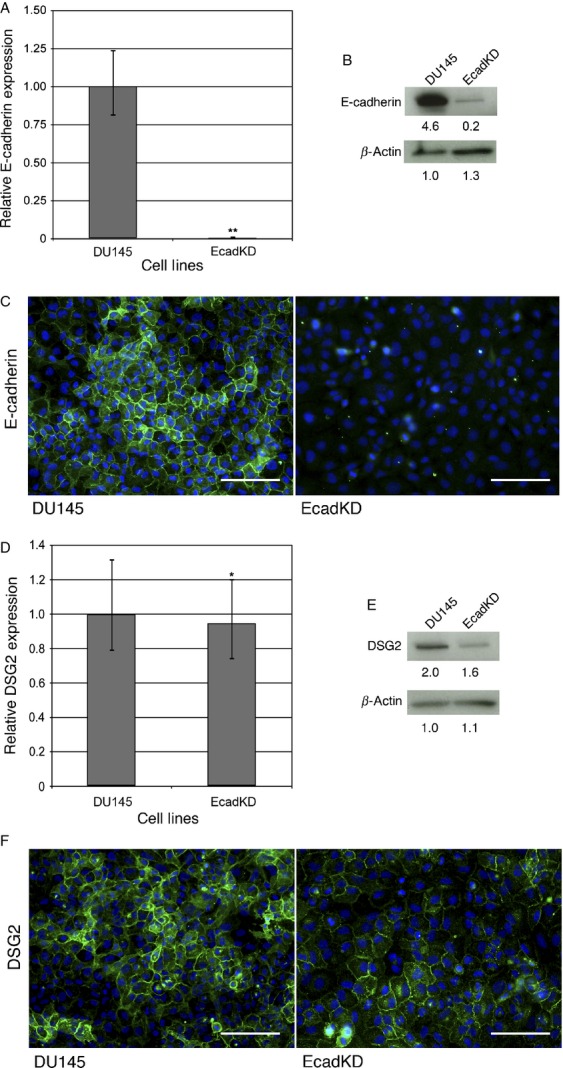
Constitutively active AKT signaling reduces E-cadherin expression via Snail transcriptional downregulation but does not affect DSG2. (A) qRT-PCR analysis shows that overexpression of constitutively active AKT results in a 93% reduction in E-cadherin mRNA expression. (B) Western Blot analysis shows that E-cadherin protein expression is dramatically reduced in the MAH cell line. (C) Representative immunofluorescence (IF) analysis shows that E-cadherin is undetectable at the cell–cell border in MAH cell line. E-cadherin is shown in green, HA in red and DAPI in blue. (D) qRT-PCR analysis shows that *DSG2* mRNA expression is slightly increased (1.6X) in the MAH cell line. (E) Western Blot analysis confirms an increase in DSG2 protein expression. (F) IF analysis shows that DSG2 is detected at the cell–cell border in MAH cells, though less frequently than as compared to the DU145 parental cell line by observation. Most of the cells that express DSG2 at the cell border also express HA, although scattered cells with high HA expression displayed loss of DSG2 (white arrows). DSG is shown in green, HA in red and DAPI in blue. (G) qRT-PCR analysis shows a slight but significant increase (1.8X) in *Snail* mRNA expression in the MAH cell line. (H) Western Blot analysis shows that Snail protein expression is comparable to that of the DU145 parental cell line. (I) IF analysis shows a dramatic increase in the nuclear localization of Snail in the MAH cell line, indicative of Snail activity. Snail is shown in red and DAPI in blue. *P <0.05; **P <* *0.01; Scale bars correspond to 100 *μ*m.

Having found that reduced expression of E-cadherin and DSG2 are associated with BCR, we next wanted to examine the mechanisms by which E-cadherin and DSG2 expression may be reduced in prostate cancer. Given the interdependence of anchoring junction assembly and regulation, to test whether loss of E-cadherin based adherens junctions results in the reciprocal loss of desmosomal adhesion, we generated a transgenic DU145 cell line that stably expressed a shRNAmir-E-cadherin construct and showed a 99.6% reduction in mRNA E-cadherin expression (referred as EcadKD; Fig.2A). This reduction was confirmed at the protein level, both by western blot (Fig.2B) and IF (Fig.2C). Interestingly, despite the dramatic reduction in E-cadherin expression, the mRNA levels of *DSG2* were relatively unchanged, showing only a slight reduction (5.3%) as compared to the parental line (Fig.2D). Consistent with the qRT-PCR findings, DSG2 protein expression was slightly reduced by western blot (Fig.2E) and cell border expression was diffusely detected in the EcadKD cell line (Fig.2F). These findings suggest that the formation of desmosomes in prostate cancer is not dependent upon the prior formation of adherens junctions.

### AKT signaling activation results in E-cadherin repression whereas DSG2 is not affected

**Figure 3:**
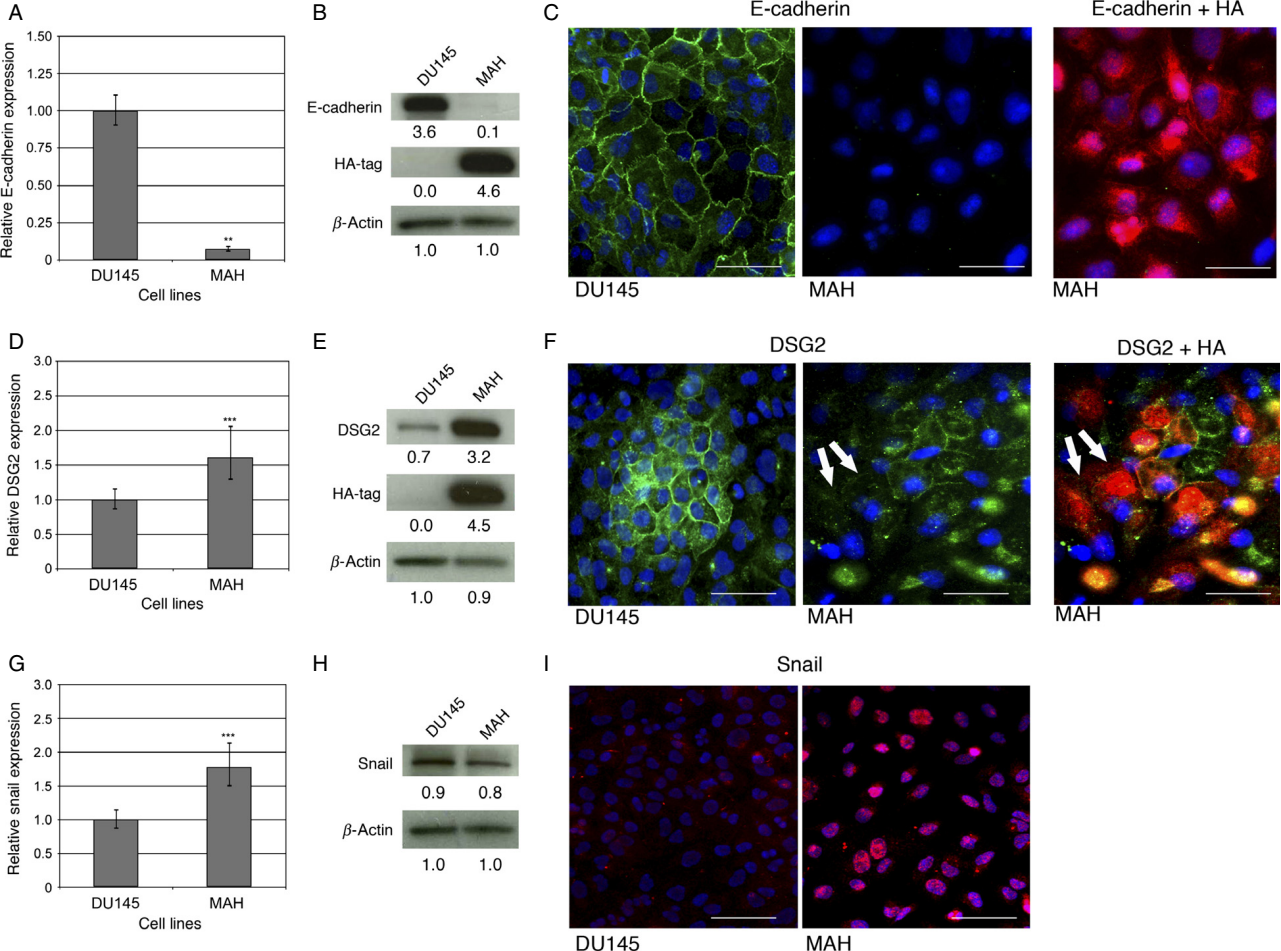
In vivo tumorigenesis assay of EcadKD and MAH cell lines. (A) Representative subcutaneous tumors generated in NOD/SCID mice after the injection of 1 × 10^6^ cells from DU145 parental cell line (white arrow), MAH cell line (black arrow), and EcadKD cell line (red arrow). (B–C) The DU145 parental cell line (left tumor) produced large tumors, whereas the MAH cell line (middle tumor) and EcadKD cell line (right tumor) produced tumors that were significantly smaller. (D) Representative immunofluorescence (IF) analysis shows high level of E-cadherin expression in tumors generated from DU145 parental cell line and MAH cell line, however, only small areas of faint, irregular expression could be detected in EcadKD tumors. Strong expression of DSG2 could be detected in all tumors examined. (E) Representative co-IF analysis of E-cadherin and HA, as well as DSG2 and HA in tumors generated from the MAH cell line. The expression of E-cadherin appears to be inversely proportional to that of MAH. The expression of DSG2 also appears to be inversely proportional to HA, though to a lesser extent than that of E-cadherin. E-cadherin and DSG2 are shown in green, HA in red, and nuclei labeled with DAPI in blue. **P < 0.01; ***P <* *0.001. Scale bar corresponds to 100 *μ*m.

As loss of adherens junctions does not lead to the reciprocal loss of desmosomes in prostate cancer in vitro, we next examined the effects of PI3K/AKT signaling on anchoring junctions in prostate cancer as this pathway has been shown to lead to the downregulation of E-cadherin and mislocalization of desmosomal proteins in squamous cell carcinoma lines 29. To activate the PI3K/AKT signaling pathway, a construct containing a myristoylated form of AKT (myr-Akt) that is HA-tagged (hereafter referred to as MAH) was overexpressed in DU145 cells 35. As expected, the MAH cell line displayed high and homogeneous levels of MAH expression (Fig.3C and 3F, right panels). Interestingly, the levels of E-cadherin were significantly reduced in the MAH cell line both at the transcript (Fig.3A, reduction of 93%, *P* < 0.01) as well as the protein level (Fig.3B–C), suggesting that AKT signaling results in the transcriptional repression of E-cadherin.

In contrast, a small but significant increase in *DSG2* was detected in the MAH cell line at the transcriptional level (1.6X, *P* < 0.001; Fig.3D) as well as at the protein level by western blot (Fig.3E). While the expression of DSG2 could be detected at the cell border of the MAH cells by IF, this localization was found in fewer cells of the MAH cell line as compared to the DU145 parental cell line (Fig.3F). Using co-IF, DSG2 cell border expression was detected in cells expressing high levels of activated AKT, however, cells with activated AKT in which DSG2 expression was either low or absent could be detected on occasion (Fig.3F, white arrows). This pattern of DSG2 expression differs greatly from that of E-cadherin and may signify that the effect of activated AKT-mediated signaling on DSG2 expression is context dependent, and different from E-cadherin.

### Activated AKT may inhibit E-cadherin via snail in prostate cancer

Having found that the expression of activated AKT results in decreased E-cadherin expression at the mRNA level, the possibility that this transcriptional repression of E-cadherin may be mediated by the EMT-associated transcription factor Snail was then examined. MAH cells showed a small but significant increase in *Snail* expression (1.8X, *P* < 0.001; Fig.3G). Interestingly, whereas western blot analysis showed that the overall level of Snail expression was comparable to that of the DU145 parental cell line (Fig.3H), there was a dramatic increase in the nuclear localization of Snail in the MAH cell line by IF (Fig.3I). Taken together these results suggest that activated AKT expression results in the nuclear accumulation of Snail and the transcriptional downregulation of E-cadherin.

### Loss of E-cadherin significantly reduces prostate cancer tumorigenesis and impairs extravasation and metastatic tumor colony formation in vivo

**Figure 4:**
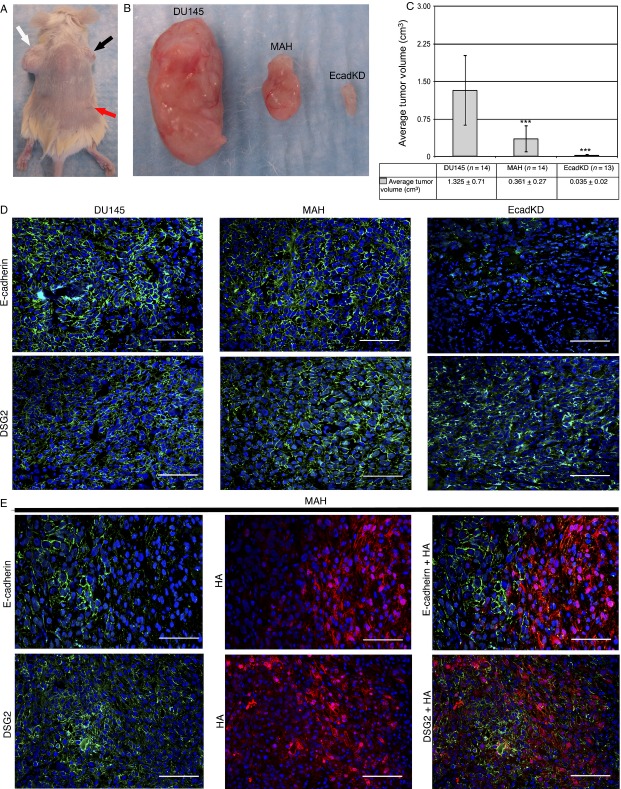
In vivo extravasation and metastatic tumor colony formation assay. (A) Animals injected with the parental DU145 cell line form large tumors in the lung parenchyma, with higher frequency. (B) Animals injected with the MAH cell line form tumors that are smaller than those formed by the DU145 parental cell line. (C) Animals injected with the EcadKD cell line form the smallest tumors observed (arrows point to tumors, red arrow indicates the tumor shown in the right panel). (D) Table summarizes the in vivo extravasation and metastatic tumor assay results. Scale bars correspond to 25 *μ*m in low magnification microphotographs (left panels) and to 300 *μ*m high magnification microphotographs (right panels). ***P < 0.001.

We next conducted an in vivo tumorigenesis assay to examine the effects of the loss of adherens junctions, as well as the effects of AKT-mediated signaling on anchoring junctions in tumor formation. Following subcutaneous injection of 1 × 10^6^ cells for our in vivo tumorigenesis assay, there was a striking and significant difference between the size of the tumors formed by the parental DU145 cell line and those formed by both the MAH and EcadKD cell lines (Fig.4A–C). Tumors generated from the MAH (0.36 ± 0.27 cm^3^; *P *< 0.001) and the EcadKD cell line (0.04 ± 0.02 cm^3^; *P *< 0.001) were significantly smaller than those generated from the DU145 parental cell line (1.33 ± 0.71 cm^3^; Fig.4B–C). Moreover, MAH tumors were significantly larger than those formed by the EcadKD cell line (*P *< 0.001).

In concordance with the in vitro profile, the DU145 parental cell line formed tumors with robust expression of both E-cadherin and DSG2 at the cell–cell border and as expected, DSG2 could also be detected at the cell border of tumors generated from MAH and EcadKD cells (Fig.4D). E-cadherin was absent from the EcadKD tumors. Interestingly, while the expression of E-cadherin was significantly reduced in MAH cell in vitro, E-cadherin was widely detected at the cell border in tumors formed by MAH cells (Fig.4D, middle). Co-IF analyses revealed that E-cadherin expression inversely correlated with the expression of MAH (Fig.4E, upper panels). Notably, this pattern of expression was also detected for MAH and DSG2, though to a lesser extent, as DSG2 expression was more robust in cells with low or no MAH expression (Fig.4E, lower panels).

**Figure 5:**
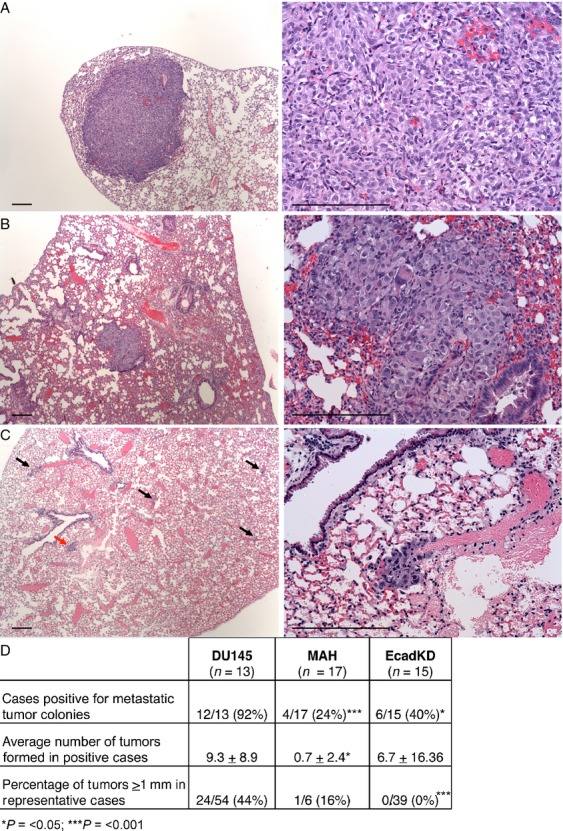
Immunofluorescence (IF) analysis of E-cadherin and DSG2; clinical implications. (A) Representative IF expression of E-cadherin in a tissue microarray (TMA) core, showing that E-cadherin expression is high in well-differentiated areas of the tumor (inside dotted line) and low in poorly differentiated areas of the tumor. (B) Representative IF expression of DSG2, displaying lower DSG2 expression in poorly differentiated areas of the tumor when compared to rare normal prostatic glands. (C) Kaplan–Meier survival curve showing that patients expressing ≥75% E-cadherin had a statistically significant longer recurrence-free survival than those expressing lower levels of E-cadherin. (D) Combined loss of DSG2 and E-cadherin is a significant prognostic marker of shorter biochemical recurrence-free survival. E-cadherin and DSG2 are shown in red, CK8/18 is shown in green, and DAPI is show in blue. Scale bar corresponds to 100 *μ*m.

We then performed an in vivo extravasation and metastasis formation assay with the same cells as described above. The amount of animals that developed metastatic tumor colonies was significantly lower for those injected with the EcadKD cell line (40%, *P* < 0.05) and MAH cell line (24%, *P* < 0.001) as compared to those injected with the parental line (92%) (Fig.5). Additionally, significantly fewer tumors formed in the animals that were injected with MAH cells (0.7 ± 2.4, *P* < 0.05) as compared to the DU145 parental cell line (9.3 ± 8.9) (Fig.5D). Although the difference between the number of tumors generated in animals injected with the EcadKD cell line versus the DU145 parental cell line was not significant, there was a striking difference in the size of these tumors (Fig.5). DU145 parental cells developed much larger metastatic tumor colonies (Fig.5A) than MAH cells (Fig.5B) and EcadKD cells (Fig.5C), the latter of which formed small metastatic tumor colonies, some comprised of no more than ten cells (Fig.5C, arrows). To provide perspective on tumor size, a cut-off of 1 mm in diameter was chosen. This analysis showed 44% of the tumors formed from the parental DU145 cell line measured ≥1 mm, whereas only 16% of the tumors from MAH cells measured ≥1 mm, and no tumor identified in the EcadKD model had a diameter ≥1 mm (Fig.5D). These results support the hypothesis that loss of E-cadherin results in the impaired development of primary and metastatic tumor colonies. Taken together they strongly suggest that high levels of PI3K/AKT signaling lead to reduced E-cadherin expression which dramatically impairs prostate tumorigenesis and metastatic development. Additionally, the retained expression of DSG2 in all the examined tumors suggests that the presence of DSG2-based desmosomal adhesion alone is not sufficient to support prostate tumor formation.

## Discussion

The challenge of prostate cancer diagnosis and treatment lies in predicting aggressive cancer. There is a great need for prognostic biomarkers that can assist in estimating the likelihood of prostate cancer aggressiveness. Though the reduction in E-cadherin expression has been reported in primary prostate cancer, no large-scale study has yet examined the association of E-cadherin with disease outcomes. We have recently reported that reduced DSG2 expression is an independent biomarker associated with a shorter BCR in prostate cancer 15. Given that both adherens junctions and desmosomes are involved in cell–cell adhesion in prostatic epithelium, an understanding of the expression of these cadherins would provide a deeper insight into the role of anchoring junctions in prostate cancer progression. In line with previous reports, we found that E-cadherin expression was significantly reduced in prostate cancer and we observed a negative correlation between the expression of both E-cadherin and DSG2 and serum PSA concentration, Gleason score, and pathological stage. Moreover, these results highlight a potentially critical role for cadherin based cell–cell adhesion in the progression of prostate cancer to a metastatic state, and demonstrate that these cadherins may be useful prognostic markers of aggressive prostate cancer.

The role of E-cadherin as a tumor suppressor has been well established by the frequently observed loss of E-cadherin in a variety of cancers as well as the results of in vivo and in vitro analyses highlighting the role of E-cadherin as an inhibitor of invasive cancer 36–39. Indeed the results of the clinical analysis of E-cadherin expression in primary prostate cancer performed in this study support this well established role of E-cadherin as a tumor suppressor. However, the robust expression of E-cadherin in hormone-refractory metastatic prostate tumors has also been reported, suggesting an alternative role for E-cadherin as a putative tumor promoter at certain points in the metastatic pathway 13. The results of our in vivo analyses support this alternative role for E-cadherin as a tumor promoter. In our tumorigenesis and extravasation and metastatic tumor colony formation assays, animals injected with EcadKD cells consistently formed significantly smaller tumors as compared to those injected with parental DU145 cells. These results demonstrate that the permanent loss of E-cadherin expression in prostate cancer cells results in impaired tumorigenesis and metastatic tumor colony formation in vivo.

The ostensibly contradictory results found in our clinical analysis versus those of our in vivo analyses suggest that E-cadherin may have a dual role in cancer progression. The duality of the role of E-cadherin as both a tumor suppressor and a putative tumor promoter may be maintained via a transient means of E-cadherin repression in prostate cancer. While the permanent loss of E-cadherin through such mechanisms as LOH coupled with inactivating mutations has been documented in gastric cancer and lobular breast carcinoma, the loss of E-cadherin expression in cancer is most often associated with impermanent mechanisms such as transcriptional silencing, transcriptional repression, or posttranscriptional modifications 40–44. One such mechanism by which E-cadherin may be transiently repressed in cancer is by epithelial mesenchymal transition (EMT), as hallmarks of EMT include reversibility and the downregulation of E-cadherin. Snail has been associated with the downregulation of E-cadherin in breast and colorectal cancer 21,22,24,25,30, results that are in concordance with our in vitro findings in prostate cancer cells. We observed that the stable overexpression of activated AKT was associated with a significant reduction in E-cadherin expression as well as a dramatic increase in the nuclear accumulation of Snail. Furthermore, the in vivo extravasation and metastatic tumor colony formation assay showed that significantly fewer animals injected with activated AKT expressing cells formed metastatic tumor colonies as compared to the control animals, and those animals that did form tumors formed significantly smaller tumors than those found in the control animals. These results demonstrate that activated AKT expression can negatively regulate E-cadherin expression resulting in impaired metastatic tumor colony formation. Moreover, as PI3K/AKT signaling has been previously associated with EMT-like events in cancer, these results implicate PI3K/AKT signaling as a candidate for an EMT-like transient repression of E-cadherin in prostate cancer via the activation of Snail 26–29,45–49.

Unexpectedly, tumors formed by activated AKT expressing cells showed E-cadherin expression, which was inversely correlated with activated AKT expression. This observed sensitivity of E-cadherin expression to the level of activated AKT expression may represent a means of regulating the inhibition of E-cadherin expression by the PI3K/AKT signaling pathway such that a certain threshold of PI3K/AKT signaling may be required for the repression of E-cadherin. The idea that certain cellular outputs are dependent upon achieving a particular AKT signaling threshold is supported by the findings of Segrelles et al. who examined the ectodermal development of myr-Akt transgenic mice displaying different levels of Akt kinase activity 50. As PI3K/AKT signaling is involved in a multitude of processes in the progression of cancer, and our results indicate that the loss of E-cadherin is only advantageous to cancer progression at some points of the metastatic pathway, the observed sensitivity of E-cadherin expression to a high threshold of activated AKT expression may represent a means of fine tuning the negative regulation of E-cadherin by PI3K/AKT signaling.

Contrary to what we observed in the clinical samples, loss of E-cadherin expression in prostate cancer cells in vitro did not alter the expression of DSG2, suggesting that the loss of E-cadherin based adherens junctions in prostate cancer does not result in the reciprocal loss of desmosomes, and that the formation of desmosomes does not strictly require the presence of adherens junctions.

Additionally, our in vitro analysis shows that the expression of *DSG2* was relatively unaffected by the homogeneously high level of AKT expression and the nuclear accumulation of Snail. Although the high levels of DSG2 expression detected in MAH cells suggested that activated AKT expression does not affect overall DSG2 protein expression, the reduced cell border localization of DSG2 suggests that activated AKT may impair desmosome formation. Thus separate pathways may be involved in the regulation of E-cadherin and DSG2 expression in prostate cancer. In summary, these results suggest that the regulation of DSG2 expression in prostate cancer is independent from that of E-cadherin.

## Supporting Information

**Figure S1.** Schematic experimental design. To assess the tumor initiation capacity of the different cell lines, 1 × 10^6^ cells from DU145 parental, myristoylated HA-tagged AKT1 (MAH) and E-cadherin knock-down (EcadKD) cells were subcutaneously injected in the upper-left flank, the upper-right flank and the lower-right flank, respectively.

**Table S1.** qRT-PCR primers. **Table S2.** Clinico-pathological features of patients (*n *=* *414)*. **Table S3.** REporting of tumors MARKer prognostic studies.
