# Supplementary figures and images for: PI3K/AKT pathway regulates E-cadherin and Desmoglein 2 in aggressive prostate cancer
Source: Cancer Med. 2015 May 29;4(8):1258–71. doi: 10.1002/cam4.463 (PMC4559037; doi:10.1002/cam4.463)

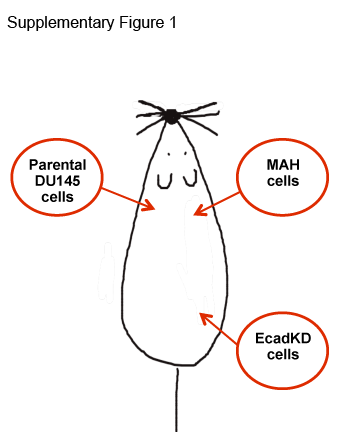

Supplement: Supplementary file 1 [file cam40004-1258-sd1.tif]
