# Supplementary material for: PI3K/AKT pathway regulates E-cadherin and Desmoglein 2 in aggressive prostate cancer
Source: Cancer Med. 2015 May 29;4(8):1258–71. doi: 10.1002/cam4.463 (PMC4559037; doi:10.1002/cam4.463)
Supplement: Supplementary file 2 [file cam40004-1258-sd2.doc]

**Supplementary data**

**Supplementary Figure Legends**

**Supplementary Figure 1. Schematic experimental design.** To assess the tumor initiation capacity of the different cell lines, 1X10^6^ cells from DU145 parental, myristoylated HA-tagged AKT1 (MAH) and E-cadherin knock-down (EcadKD) cells were subcutaneously injected in the upper-left flank, the upper-right flank and the lower-right flank, respectively.

**Supplementary Tables**

**Supplementary Table 1. qRT-PCR Primers**

| qRT-PCR Primers | | |
| --- | --- | --- |
| Transcript | Forward Primer | Reverse Primer |
| E-cadherin* | 5-’CAGCACGTACACAGCCCTAA-3’ | 5’-ACCTGAGGCTTTGGATTCCT-3’ |
| DSG2 | 5’-ATCAATGCAACAGATGCAGATGA-3’ | 5’-TGTCAAAGTGTAGCTGCTGTGT-3’ |
| Snail* | 5’- CTTCCAGCAGCCCTACGAC-3’ | 5’-CGGTGGGGTTGAGGATCT-3’ |
| β-actin | 5’-AAACTGGAACGGTGAAGGTG-3’ | 5’-GTGGCTTTTAGGATGGCAAG-3’ |

*E-cadherin and Snail primers designed (1).

**Supplementary Table 2:** **Clinico-pathological features of patients (*n*=414)***

| **Age at Diagnosis** |  |
| --- | --- |
| Mean | 61 |
| Range | 41-74.7 |
| Unavailable | 5 (1%) |
|  |  |
| **Race** |  |
| White | 230 (56%) |
| African American | 179 (43%) |
| Asian/Polynesian | 1 (<1%) |
| Hispanic | 2 (<1%) |
| Unknown | 2 (<1%) |
|  |  |
| **PSA at Diagnosis (ng/mL)** |  |
| Mean | 7 |
| Range | 0.4-51.4 |
| <4 | 72 (17%) |
| 4 - 10 | 272 (66%) |
| >10 | 66 (16%) |
| Unavailable | 4 (<1%) |
|  |  |
| **Gleason Score** |  |
| ≤ 6 | 148 (36%) |
| 7 | 177 (43%) |
| 8 | 52 (13%) |
| ≥ 9 | 25 (5%) |
| Unavailable | 12 (3%) |
|  |  |
| **TNM Stage** |  |
| T1, T2 | 329 (79%) |
| T3 | 73 (18%) |
| T4 | 10 (2%) |
| Unavailable | 2 (<1%) |
|  |  |
| **Follow-up (months)** |  |
| Mean | 56.8 |
| Range | 1.4-123.6 |
|  |  |
| **Biochemical Relapse** |  |
| Negative | 316 (76%) |
| Positive | 98 (24%) |

*As reported in (15)

**Supplementary Table 3. REporting of tumors MARKer prognostic studies.**

| **Introduction** |
| --- |
| We hypothesized that loss of cadherins may be associated with aggressive prostate cancer. Thus, E-cadherin and DSG2 expression was assessed in Tissue Microarrays (TMAs) containing prostate cancer tissues by Immunofluorescence. |
| **Material and Methods** |
| **Patients:** This study focused on the analysis of formalin-fixed paraffin embedded (FFPE) tissue samples obtained from prostatectomy specimens of 414 patients with a diagnosis of primary prostate cancer. Follow-up data [mean follow-up: 56.8 months (range: 1.4 - 123.6 months)] for all the cases regarding evidence of biological recurrence was available for this study. These cases were originally collected between September 2000 and January 2005 at the Henry Ford Health System in Detroit, following an Institutional Review Board’s approved protocol #1018.  **Prostate cancer tissue analysis:** TMAs were built as follows: Hematoxylin & Eosin stained sections from FFPE prostatectomy specimens were reviewed to identify viable, morphologically representative areas of normal prostate glands and acinar adenocarcinoma from which needle core samples could be taken. From each specimen, triplicate adjacent tissue cores with diameters of 0.6mm were punched and arrayed onto a recipient paraffin block using a precision instrument (Beecher Instruments, MD). All punches were obtained from a focus with the highest Gleason Score, and the majority of punches were taken from the dominant tumor nodule — defined as the largest nodule with the highest Gleason Score and stage in a specific prostatectomy specimen. Consecutive five-micrometer sections of these TMA blocks were used for immunofluorescence analysis, and the first section was stained with Hematoxylin & Eosin to serve as a template for morphology.  **Immunofluorescence detection and evaluation:** The slides were deparaffinized and rehydrated, and antigen retrieval was performed by heating slides in a steamer in citrate buffer, pH 6.0 for 15 minutes. Slides were then incubated in 0.1% Triton X-100/ 1% BSA/ 1X PBS blocking serum at room temperature for 1 hour followed by primary antibody incubation overnight at 4ºC. Antibodies used correspond to: anti-E-cadherin (mouse monoclonal; Invitrogen, Carlsbad, CA), anti-DSG2 (mouse monoclonal (DG3.10), and anti-CK8/18 (guinea pig polyclonal; Progen, Heidelberg, Germany). The following day, slides were washed and then secondary antibody, either Alexa Fluor® 594 or Alexa Fluor® 488 (Invitrogen, Carlsbad, CA), was added and slides were incubated at room temperature for 45 min. Slides were then washed and mounted using VECTASHIELD® mounting medium with DAPI (Vector Laboratories, Burlingame, CA). CK8/18 expression was used to identify all epithelial areas (both tumor and normal glands) in the cores and the expression of the proteins of interest was scored by determining the percentage of tumor cells |
| with immunoreactivity per tissue core (from 0% to 100%). The evaluation was performed by a uropathologist (MCM) following previously used methodology: tumor areas in the core were first identified by DAPI, confirmed by the presence of CK8/18 and then scored by establishing a percentage of tumors cells with membrane expression of E-cadherin and DSG2. The average values of the representative cores from each patient sample were then used for statistical analyses. A positive cut-off of 75% and 60% were used to perform statistical analyses of correlation with clinico-pathological features and survival as these were the approximate median expression values observed for E-cadherin and DSG2 in this prostate cancer cohort, respectively.  **Clinical endpoints:** Biochemical recurrence was defined as a post-surgery undetectable PSA reading followed by two consecutive detectable (>0.2 ng/ml) rising PSA levels four weeks or more post-surgery (3).  **Sample size determination:** A sample size calculation was performed for the differential expression of 2 molecular markers across the patients, which suggested a minimum sample size range of 103 to 148 samples.  **Statistical analyses:** Statistical analyses were conducted using SPSS v20.0 (IBM, Chicago, IL). Biochemical recurrence (BCR) free survival was analyzed using Kaplan-Meier survival curves, and compared using the log-rank test. A two-sided *P*-value ≤ 0.05 was considered statistically significant. |
| **Results** |
| **Relationship to standard prognostic variables:** Correlation of E-cadherin with these variables is summarized in Table 2. We observed a negative correlation between the studied biomarker and Gleason Score, pathological stage and pre-surgical PSA, although only the first was statistically significant.  **Analyses of biomarkers’ expression:** From the cases analyzed, 152 tumors (47.4%) showed reduced expression of E-cadherin (expression in less than 75% of the tumor cells), and these patients presented with a worse clinical outcome, with a shorter biochemical recurrence free survival time. Multivariate analyses including showed that only DSG2 was a significant independent prognostic factor in our cohort, as previously reported in 4. |
| **Discussion** |
| **Conclusions:** Our findings confirm our hypothesis that reduced expression of cadherins is observed in patients with a shorter biochemical recurrence free survival.  **Clinical value:** The identification of these biomarkers as independent prognostic factors could be used to determine the prognosis of a patient at the moment of diagnosis, to assess his personalized clinical management. However, further studies in prostate biopsies to confirm this finding need to be undertaken to further support the use of these biomarkers in the clinical setting. |

**References**

1. Chen J, Imanaka N, Griffin JD. Hypoxia potentiates Notch signaling in breast cancer leading to decreased E-cadherin expression and increased cell migration and invasion. *Br J Cancer.* 2010; **102**: 351-360.
